# Design and Evaluation of a Novel Hybrid Soft Surgical Gripper for Safe Digital Nerve Manipulation

**DOI:** 10.3390/mi10030190

**Published:** 2019-03-15

**Authors:** Jin Guo, Jin-Huat Low, Yoke-Rung Wong, Chen-Hua Yeow

**Affiliations:** 1 Department of Biomedical Engineering, National University of Singapore, Singapore 119077, Singapore; biegjin@nus.edu.sg; 2 Advanced Robotics Centre, National University of Singapore, Singapore 119077, Singapore; lsiljh@nus.edu.sg; 3 Biomechanics Laboratory, Singapore General Hospital, Singapore 169856, Singapore; wong.yoke.rung@sgh.com.sg

**Keywords:** soft pneumatic actuator, surgical gripper, digital nerve manipulation

## Abstract

Forceps are essential tools for digital nerve manipulation during digital nerve repair surgery. However, surgeons have to operate forceps with extreme caution to prevent detrimental post-operative complications caused by over-gripping force. Their intrinsically safe characteristics have led to the increasing adoption of soft robotics in various biomedical applications. In this paper, a miniaturized hybrid soft surgical gripper is proposed for safe nerve manipulation in digital nerve repair surgery. This new surgical gripper includes a soft inflatable actuator and a gripper shell with a hook-shaped structure. The ability to achieve a compliant grip and safe interaction with digital nerves is provided by the inflated soft pneumatic actuator, while the rigid hook retractor still allows surgeons to scoop up the nerve from its surrounding tissues during surgery. The performance of the proposed surgical gripper was evaluated by the contact/pulling force sensing experiments and deformation measurement experiments. In the cadaver experiments, this new surgical gripper was able to complete the required nerve manipulation within the limited working space. The average deformation of the digital nerve with an average diameter of 1.45 mm gripped by the proposed surgical gripper is less than 0.22 mm. The average deformity is less than 15% of its original diameter.

## 1. Introduction

Digital nerves are the nerves that supply sensations such as pain, discriminatory touch, or other sensations between the brain and fingers [1]. They are located with the digital arteries along the sides of each finger. Digital nerves can be damaged by many different causes, including stretch and avulsion injuries (excessive strain exerted on a nerve), crush and compression injuries (external forces crushing the tissue), and penetrating injuries (shape laceration by a knife or piece of glass) [2]. In addition, iatrogenic nerve injuries caused by medical interventions or inflicted accidentally by a treating surgeon also may result in digital nerve damage [3,4]. Digital nerve repair is a microsurgical procedure used to treat a severed or damaged nerve by reconnecting the ends of the severed nerve in the hand to allow the nerve to heal, so as to reduce the possibility of scarring, neuroma, or painful growths [5,6].

**Figure 1:**
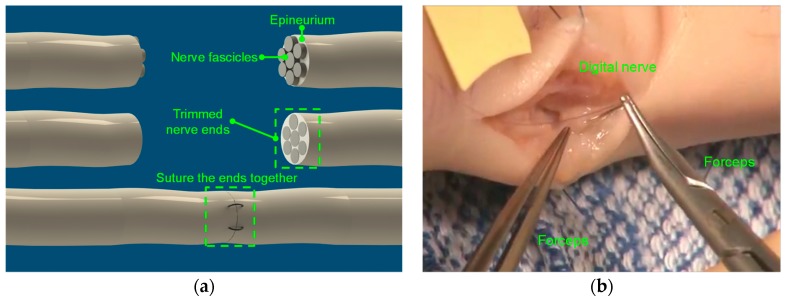
The surgical procedures of digital nerve repair: (**a**) direct repair with epineural sutures; (**b**) the forceps used in real digital nerve repair surgeries.

After the patient has been given anesthesia, sharp dissection is used to free the damaged ends of the injured digital nerve, and the surgeon examines the damaged ends of the nerve to determine the best course of action by using an operative microscope. The damaged ends of the nerve are trimmed to reveal healthy nerve fascicles, which are then repaired via end-to-end neurorrhaphy with fine sutures placed in the epineurium (shown in Figure 1a) if the ends of the nerve can be pulled together without creating tension [7,8]. The nerve conduit may be used to bridge the gap when substantial tension is required to bring the nerve ends together. The surgeon sews the ends of the nerve to the conduit ends in order to form a channel guiding the fascicles as they grow together and rejoin [9,10].

It has been reported by a research group from University of Washington [11] that the mechanical stress caused by a minimally invasive surgery (MIS) grasper can cause unintended damage to tissue by several means during tissue manipulations in MIS and even less severe immediate injury from grasping or manipulation may still lead to clinically relevant consequences. Marucci et al. [12] mentioned that iatrogenic trauma due to grasper manipulation causes major complications in laparoscopic surgery. Digital nerves are smaller and more delicate than the tissues involved in MIS. Surgeons have to be extremely cautious to prevent over-gripping damage when operating forceps in digital nerve repair surgery (as shown in Figure 1b). Only a minority group of veteran surgeons are capable of performing such surgeries due to the fear of nerve damage caused by over-gripping force during the nerve repair surgery. Regenbogen et al. [13] reviewed technical errors from 444 surgical malpractice claims from liability insurers. From their research reports, the majority of surgical adverse events involved technical errors and two-thirds of the technical errors were linked to manual error. In addition, a majority of the technical errors involved routine operations and 73% involved experienced surgeons operating within their area of expertise and training. Thus, there is a strong drive to design a new surgical gripping technology for safe digital nerve manipulation.

Soft-bodied robots made of soft materials can perform better in the interaction with organs and tissues than instruments with rigid structures, because they can deform and absorb much of the energy arising from a collision [14,15,16,17]. Regarding the existing soft surgical grippers, Liang et al. [18] developed a micro, soft pneumatic actuator inspired by shape engineering and validated its future potentials in biomedical applications by wrapping the sciatic nerve of a rat. This actuator can wrap around the nerve tissues but cannot grip the nerves firmly. Lu and Kim et al. [19,20] proposed a micro-gripper for retinal surgery. The micro-gripper can hold multiple micro-fingers according to different applications, and each finger is composed of silicon phalanges connected by the joints (polymer balloon actuators). The maximum gripping force generated by the micro-gripper with four micro-fingers is approximately 20 mN at 551 kPa. Another similar soft robotic actuator was presented by Gorissen et al. [21]. The maximum gripping force based on these two soft actuators was reported to be 44 mN at 105 kPa. Konishi et al. [22] developed a novel surgical tool for retinal pigment epithelium (RPE) sheet transplantation based on the pneumatic balloon actuator. The largest force generated by the transplantation tool is about 3 mN. Tweezer-like soft surgical grippers and a three-bloat soft surgical gripper were developed to allow for compliant gripping of small objects [23,24]. However, the gripping components are bulky, which encroaches on the limited available space for the suturing of the digital nerves. In addition to the surgical application, soft grippers have been widely applied in delicate object grasping applications. A fluid-driven micro bio-gripper and piezoelectric material-based micro gripper were presented in [25,26], respectively. In addition, self-folding thermos-magnetically responsive soft microgrippers were proposed by a research group from the Johns Hopkins University in [27]. A passive compliant robotic gripper embedded with sensors made by conductive silicone rubber elements and an adaptive neuro fuzzy inference strategy for controlling the input displacement of the compliant gripper were proposed by Petković et al. [28]. However, these designs are difficult to extend to the nerve manipulation in digital nerve repair surgery due to the large prototype size or limited gripping force.

In addition to soft gripping the nerve, a surgical gripper should have the ability to scoop up the nerve from the surrounding tissues, which include muscle, blood vessels, and connective tissues. In this paper, a hybrid soft surgical gripper, which combines both a soft gripping component and a rigid nerve hook retractor, is proposed for safe digital nerve manipulation in digital nerve repair surgery. The proposed surgical gripper allows for the scooping up of the digital nerves by the hook-shaped structure and then offers a compliant grip and soft contact with digital nerves via the inflated portion of the soft pneumatic actuator when pressurized air is applied. To the best of our knowledge, no surgical grippers combining soft pneumatic actuators for digital nerve manipulation have been reported. The merits of the proposed surgical gripper are summarized as follows: (1) compared with forceps with rigid structures, the proposed surgical gripper can offer a compliant grip and soft contact with digital nerves provided by the inflated soft pneumatic actuator; (2) the inflated soft pneumatic actuator of the proposed surgical gripper can provide a steady gripping force when constant pressurized air is supplied. In addition, it can deform and absorb much of the energy arising from a collision. The proposed surgical gripper can avoid unintended excessive stress damage to digital nerves while handling nerves using forceps requires extreme caution; and (3) compared with the existing soft surgical grippers, the proposed surgical gripper can provide much greater gripping force, and it still allows surgeons to scoop up the nerves from the surrounding tissues.

## 2. Materials and Methods

### 2.1. Target Application and Performance Requirements

The soft surgical gripper presented here is designed for nerve manipulation in digital nerve repair surgery. During the surgical procedures, the surgeon will operate the soft surgical gripper to grip and hold the ends of the damaged digital nerve for neurorrhaphy and to rotate the gripped nerve for observation. The performance requirements for the proposed surgical gripper are as follows:

(1) Overall surgical gripper size: The length of an incision made over the lesion on the patient’s finger is approximate 2 cm, and the required working space for end-to-end neurorrhaphy is estimated at approximately 10 mm. The size of the new surgical gripper must be determined to guarantee that the new gripper can complete the required nerve manipulation within the limited working space;

(2) Deformation: Although there is lack of in-vivo research to describe the maximum tolerance of digital nerve deformation, it is believed that the neurons can be compressed too far and become dysfunctional. Therefore, the new surgical gripper aims to achieve minimal deformation of the digital nerve during repair surgery;

(3) Gripping force range: The required gripping force range is approximately 0–1.0 N based on a summary of reported microsurgical force characterization experiments, including brain surgeries, retinal surgeries, and small bold vessel anastomosis [29,30]. It is reported that the maximum gripping force seen in microsurgical manipulation is no more than 1.0 N.

### 2.2. Prototype of the Proposed Surgical Gripper

**Figure 2:**
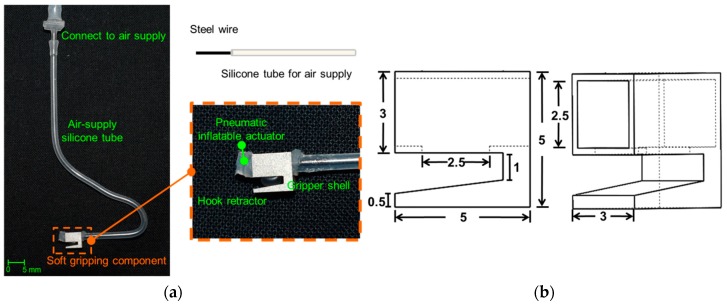
A prototype of the hybrid surgical gripper for nerve manipulation: (**a**) the soft gripping component; (**b**) the size of the prototype (dimensions in millimeters).

The proposed surgical gripper (as shown in Figure 2a) consists of a soft inflatable actuator, a stainless-steel gripper shell, and a silicone air-supply tube. The size of the whole surgical gripper is 5 mm × 5 mm × 3 mm, and the nest for the pneumatic inflatable actuator is 2.5 mm × 2.5 mm × 5 mm (as shown in Figure 2b). The opening area for the inflation of the soft pneumatic channel is 2.5 mm × 2.5 mm × 0.25 mm. The size of the new surgical gripper is determined based on the required space for neurorrhaphy according to clinical requirements. A steel wire with a diameter of 0.8 mm is inserted into the air-supply silicone tube as a flexible arm to ensure that the silicone tube still has adequate rigidity to hold the gripper in place. The flexible arm can be bent into any configurations easily, which allows the surgeon to rotate the gripper manually while holding the nerve for observation. The soft inflatable actuator is positioned inside the nest and the pneumatic channel inflates through the opening area to push the nerve against the retractor and hence achieve a compliant grip.

### 2.3. Design of the Soft Inflatable Actuators

The soft inflatable actuator is composed of a silicone tube, which is used to provide air pressure, and a pneumatic channel, which is inflated to provide the inflation to grip the nerve. Two types of soft inflatable actuators—the conventional design and the proposed pneumatic actuator—are examined and compared in this section.

**Figure 3:**
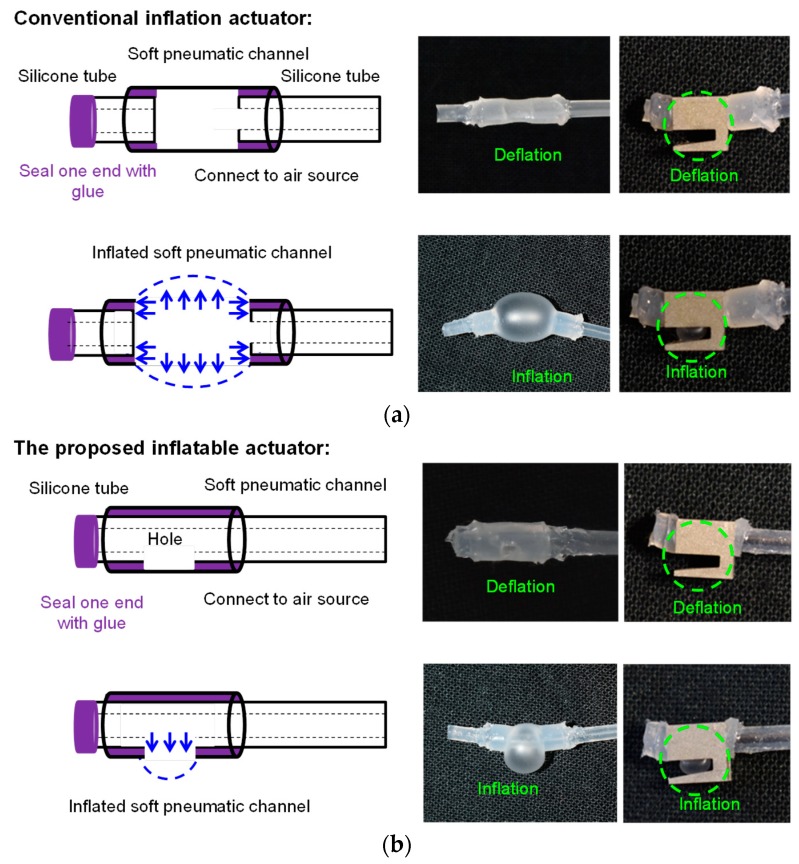
Two types of soft inflatable actuators: (**a**) the whole soft pneumatic channel is inflated by a silicone tube; (**b**) the membrane surface of the soft pneumatic channel is inflated by a hole in the silicone tube.

**Figure 4:**
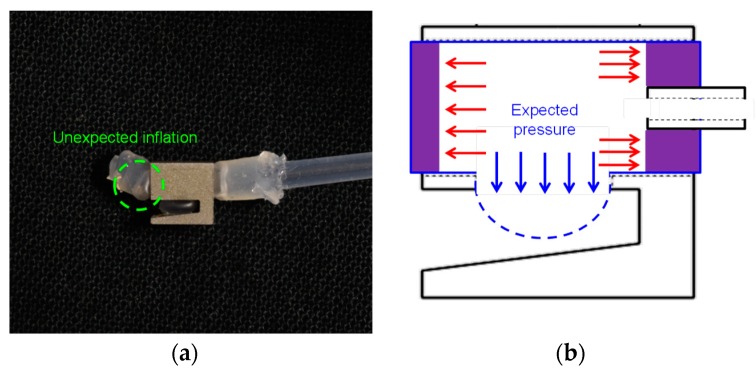
The failure mode of the conventional inflation actuator: (**a**) the unexpected inflation at the connection parts between the silicones and the soft pneumatic channel; (**b**) the cause of the unexpected inflation (the red arrows represent unexpected pressure).

In the conventional design (as shown in Figure 3a), two silicone tubes are introduced to hold the soft pneumatic channel. One silicone tube is inserted into the pneumatic channel to supply air pressure, and Sil Poxy Glue (Smooth-On, Macungie, PA, USA) is used to seal the connection part between this tube and the pneumatic channel to prevent air leakage. The other silicone tube is employed to hold and close the end of the pneumatic channel with the glue. After the soft inflatable actuator is fabricated, it is inserted into the nest of the stainless-steel shell. When air pressure is supplied to the pneumatic channel through the air-supply silicone tube, the pneumatic channel starts to inflate through the opening area at the bottom side of the nest and compress the gripped small objects. The advantage of the conventional design is that it is much easier to obtain large inflation with less air pressure. However, unexpected inflation (as shown in Figure 4a) occurred at the connection parts between the silicone tube and the soft pneumatic channel during our experiments when approximately 35 kPa was supplied. The cause of the unexpected inflation is shown in Figure 4b. The conventional soft inflatable actuator will inflate along all directions when pressurized air is supplied. When pressure is increasingly supplied to the pneumatic channel, the elastomer at the opening area is the first inflation part, because it requires less pressure to form a balloon. When pressure is increased, the stretching motion of the pneumatic channel is actuated, in addition to the inflation through the opening area. The stretching force pushes the silicone tubes out of the nest of the gripper and leads to the unexpected inflated bubble.

A new design of the soft inflatable actuator is thus proposed to eliminate unexpected inflations. Instead of using the elastomer as the body, a silicone air-supply tube is introduced to traverse through the soft pneumatic channel to avoid its stretching motion (as shown in Figure 3b). A hole is cut in the silicone air-supply tube to provide air pressure. The pneumatic channel covers the hole, and Sil Poxy glue is applied to the surrounding area of the cut and also on the opening end of the tube to seal the end. The entire inflatable actuator is then inserted into the nest of the gripper to provide inflation. When the entire structure is pressurized, the air pushes the elastomer surrounding the hole-region, and hence, inflation of the actuator occurs (as shown in the right figure of Figure 3b). The thickness of the inflatable section of the gripper is approximately 0.75 mm. Air pressure is gradually increased to the pneumatic channel, and the unexpected inflations are eliminated. This new design allows the inflatable actuator to sustain much more air pressure, thereby providing greater gripping force.

### 2.4. Fabrication of the Soft Pneumatic Channel

A communicating vessels-based fabrication method was proposed for the fabrication of the soft pneumatic channels. The fabrication procedures for the soft pneumatic channels consist of 3D-printing of the rigid components, the molding process for the soft pneumatic channels, and the detaching process.

**Figure 5:**
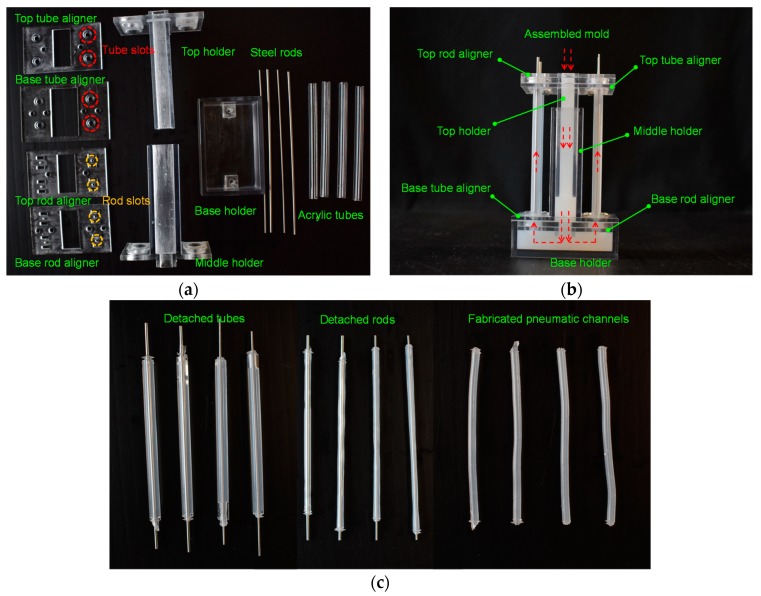
Fabrication procedures of the soft pneumatic channels: (**a**) the molds including holders, tube aligners, rod aligners, acrylic tubes, and steel rods; (**b**) the assembled mold; and (**c**) the detached tubes, detached steel rods, and the fabricated pneumatic channels.

The detailed fabrication steps are shown in Figure 5. First, the molds, including the top, middle, and base holders; tube aligners; rod aligners; acrylic tubes; and steel rods, are prepared. The holders, tube aligners, and rod aligners are 3D-printed (Stratasys, Eden Prairie, MN, USA) with VeroClear RGD 810 material. These molds are shown in Figure 5a. The tube and rod aligners are first integrated with the holders. The acrylic tubes are then fixed to the slots of the top and base tube aligners. The steel rods are inserted through the holes of the top rod aligner and locked to the holes of the base rod aligner. The slots and holes in the base and top aligners are designed to align and fix the acrylic tubes and steel rods, which can guarantee that the air channels inside the soft bodies are fabricated with consistent dimensions along the bodies. After the molds are assembled, the prepared silicone material is poured into the middle groove (shown in Figure 5b). The silicone material will gradually flow into the acrylic tubes from bottom to top based on the principle of communicating vessels until the acrylic tubes are completely filled. This procedure usually requires 5 min. The soft pneumatic channels are molded inside the acrylic tubes. Thus, the inner diameter of the acrylic tubes defines the outer diameter of the soft pneumatic channel. Steel rods are used to form air channels inside the soft cylinders. Therefore, the diameter of the steel rod determines the inner diameter of the soft pneumatic channel. The length of the fabricated soft pneumatic channels depends on the length of the acrylic tubes. The soft pneumatic channels, with different dimensions according to different applications, can be fabricated by using corresponding acrylic tubes and steel rods. In addition to acrylic tubes and steel rods, other tubes having a smooth inner surface (e.g., stainless-steel tubes) and rigid rods (e.g., carbon pins or plastic rods) holding the smooth outer surface can also be used to form the soft pneumatic channel in the fabrication process. In our case, we needed a soft pneumatic channel with a thickness of 0.75 mm. The selected inner diameter of the acrylic tubes and the diameter of the steel rods were 3 mm and 1.5 mm, respectively. The advantage of the communicating vessels-based fabrication method is that it can effectively avoid air bubbles and allow the silicone materials to enter every corner of the thin cavities. Even if there are some visible air-bubbles generated during the procedures, the upward soft material flow can push the bubbles to the top, and the main part of the soft pneumatic channel remains intact. Another notable feature of this fabrication method is that room temperature (approximately 24 °C) is used for curing instead of an oven, in which micro-bubbles may inflate and form larger bubbles when heated, which may spoil the structure of the soft pneumatic channel when pressurized air is provided. The curing procedure usually takes approximately 3 h, and the demolding process is conducted by detaching the holders, detaching the top and base aligners, taking out the acrylic tubes, and pulling out the steel rods from the soft pneumatic channels (as shown in Figure 5c). The fabrication method is very robust, and the air bubbles are eliminated from the soft bodies. The structures of the inner air channels are highly consistent along the body due to the fine inner wall of the acrylic tubes and the alignment of the rods.

After obtaining these soft pneumatic channels, the inflatable actuators can be fabricated and connected to pneumatic power according to the methods mentioned above.

### 2.5. Control System

**Figure 6:**
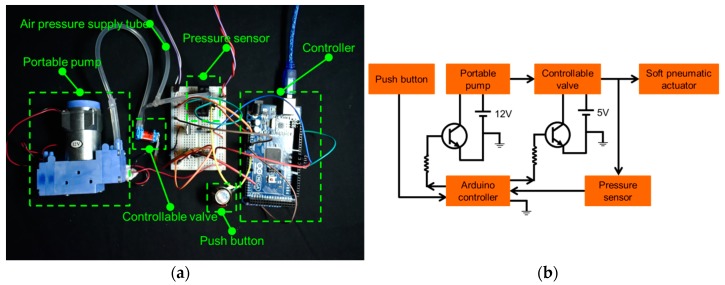
The control system of the soft hybrid surgical gripper: (**a**) the components of the control system; (**b**) the circuit diagram of the control system.

The control system of the proposed surgical gripper (as shown in Figure 6a) includes a push button, a portable pump (D737-23-01, Parker Hannifin Corporation, Mayfield Heights, OH, USA), a valve (X-Valve, Parker Hannifin Corporation), and a control board (Arduino UNO, ATmega328, company, city, state abbr. if USA or Canada). The maximum free flows of the pump and valve are both 11 slpm. When the push button is triggered, pressurized air is supplied to the soft pneumatic channel for holding the object, and once the push button is triggered the second time, the valve is activated to release the air and the gripped object. The amount of time for actuating the proposed surgical gripper is approximately 220 ms. A pressure sensor is used to monitor the input air pressure to the soft pneumatic inflatable actuator. The valve is activated to supply the air when the input air pressure is less than the required value (150 kPa in our case). On the contrary, the valve is activated to release the air when the input air pressure is more than the required value. The connection of the components in the control system is shown in Figure 6b.

## 3. Experimental Results

In order to evaluate the performance of the proposed soft hybrid robotic surgical gripper described above, three types of experiments were conducted:

(1) Force sensing experiments: to determine the relationship between the pulling/gripping forces generated by the inflated soft actuator and the input air pressure;

(2) Deformation measurement experiments: to measure the deformation of the digital nerves gripped by the proposed surgical gripper with respect to the input air pressure;

(3) Gripping tests: to demonstrate the gripper’s ability to grip the digital nerve within the required working space in digital nerve repair surgery based on cadaver experiments.

### 3.1. Force Sensing Experiments

During digital repair surgery, the gripping force must guarantee that the surgical gripper can hold the nerve firmly and rotate the nerve for observation. The relationship between the gripping force and the air pressure was determined for estimating the normal force exerted on the nerve during the surgical procedures. Additionally, the pulling force was measured using Instron Universal Tester (Instron, Norwood, MA, USA) and using real human digital nerves.

**Figure 7:**
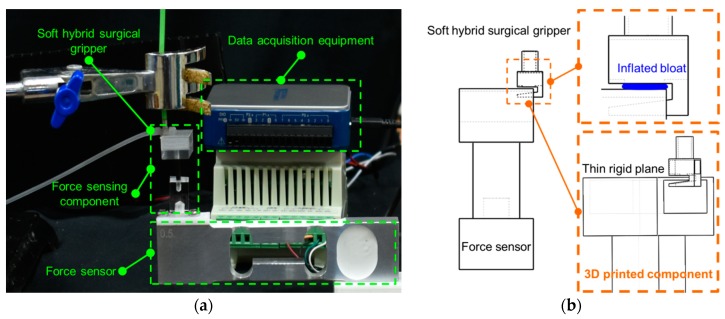
The schematic diagram of the contact force sensing experiments: (**a**) the experimental setup for measuring the force generated by the inflation of the soft pneumatic channel; (**b**) the zoom-in figure of the sensing part between the inflated bloat and the thin rigid plane.

To measure the contact force generated by the inflation of the soft pneumatic actuator inside the hook structure of the proposed soft hybrid surgical gripper, a support component (as shown in Figure 7) was 3D-printed to translate the press to a sensitive force sensor. The thickness of the thin top plane of the support component is 0.3 mm. The thin top plane was positioned in the middle of the hook structure. When the air pressure was supplied to the inflatable actuator, the inflated balloon contacted and pressed on thin top plane. The contact force could then be sensed by the force sensor.

**Figure 8:**
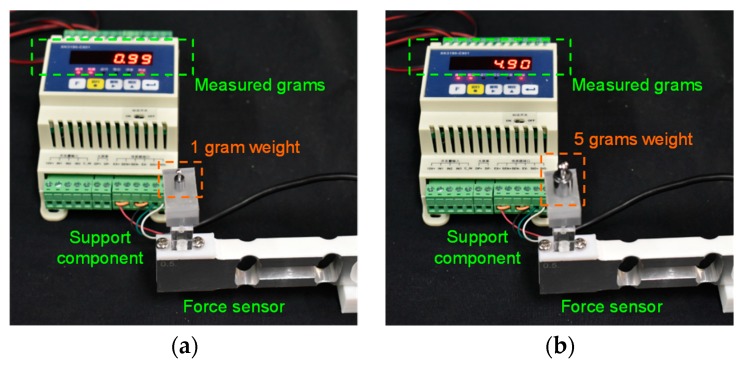
Measurement results for dead weights: (**a**) 1 g; (**b**) 5 g.

The force sensor (XK3190-C801, Shanghai Yaohua, Shanghai, China) used in this paper has a sensing range of 0–5 N, and its analog output is from 0 V to 5 V. A 16-bit analog-to-digital converter (ADC) was employed to sample the signals from the force sensor. We used several dead weights, including 1 g and 5 g to validate the accuracy of the force sensor. These weights were positioned on the thin top plane of the support component, and the measured grams were shown on the screen. The errors between the measured weight and the dead weights were less than 0.1 g, approximately equaling 0.001 N (as shown in Figure 8). Thus, the thin top plane can perceive even a very tiny compressive force from the inflation of the soft inflatable actuator accurately.

**Figure 9:**
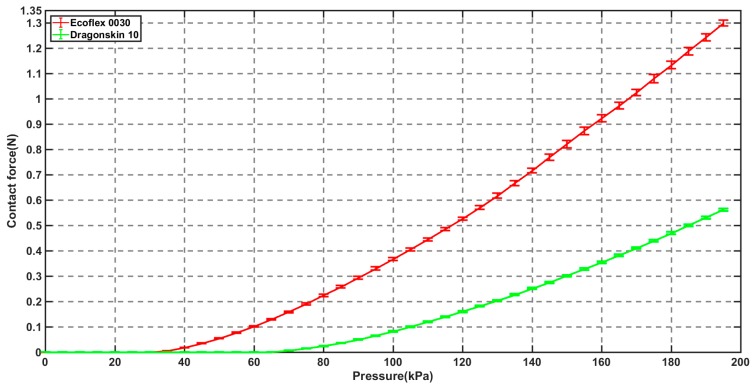
The experimental results for the measurement of the contact force.

The experimental setup for sensing the contact force generated by the soft inflatable actuator is shown in Figure 7a. A holder was used to position the hybrid surgical gripper and ensure that the thin top plane of the support component was located in the middle part of the hook structure. A pump was employed to increasingly supply pressurized air to the soft pneumatic channel to produce the inflation through the silicone tube. The pressure values and force signals were recorded by 16-bit data acquisition equipment at the same time. In order to explore which silicone material is more suitable to our application, two common types of soft materials (Ecoflex 0030 and DragonSkin 10 medium, Smooth-On, Macungie, PA, USA) with different Young moduli were used to fabricate the soft pneumatic channels based on the proposed fabrication procedures. According to the size of the nest for the soft inflatable actuator, a thickness of 0.75 mm was selected for both soft pneumatic actuators. Two types of soft inflatable actuators were used to determine the relationship between the input air pressure and contact force, respectively. The average force with deviation bars is shown in Figure 9. The contact force depends on the input air pressure and the contact area between the inflated balloon and the thin top plane. When the air pressure was gradually supplied to the soft pneumatic actuator, the balloon was expanded along the gap between the thin top plane and the opening area of the nest of the surgical gripper. The contact area was also enlarged with the increasing of the air pressure. Thus, the relationship between the contact force and the input air pressure is not linear. Based on the experimental results, the soft pneumatic actuator made of Ecoflex 0030 can generate greater force with much lower air pressure than that made of DragonSkin 10 medium. In addition, the soft actuator made of Ecoflex 0030 slightly showed Mullin’s effect after five repeated experiments, while the soft actuator made of DragonSkin 10 medium was more stable. As the soft inflatable actuator made of Ecoflex 0030 can provide over 1 N force with much smaller air pressure and its compliance is also small, Ecoflex 0030 was selected to fabricate the soft pneumatic channel of the surgical gripper for digital nerve surgery. The relationship between the contact force and the input air pressure based on the soft inflatable actuator made of Ecoflex 0030 was determined by the general Fourier-model-based fitting with a root-mean-square error (RMSE) of 0.0042. The fitting equation is as follows:

(1) $$\{\begin{array}{c} F_{\mathrm{contact}}=0,\;x\leq 34\;\mathrm{kPa}\\ F_{\mathrm{contact}}=a_{0}+a_{1}\times \cos\left(\omega \times x\right)+b_{1}\times \sin\left(\omega \times x\right),\;x>34\;\mathrm{kPa} \end{array}$$

where *a*_0_, *a*_1_, *b*_1_, and ω equal 1.439, −1.487, −0.006228, and 0.007646, respectively. An approximate contact force value can be estimated according to the current air pressure based on Equation (1) during a surgical procedure.

**Figure 10:**
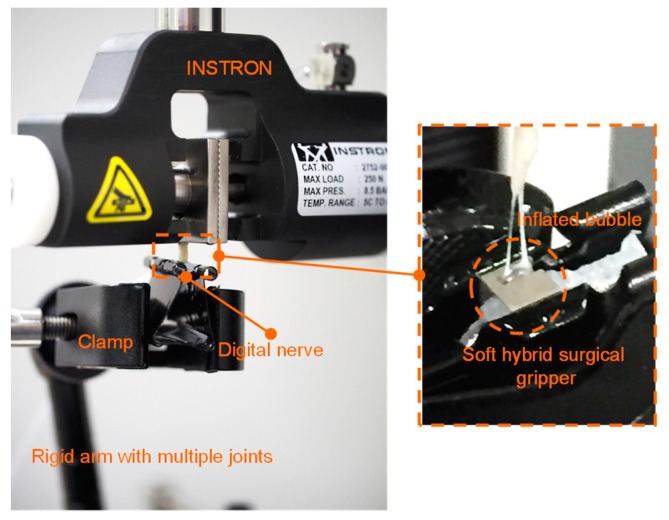
The experimental setup for measuring the pulling force based on the soft hybrid surgical gripper.

The pulling force depends on the coefficient of friction of the contacted surface between the gripped object and the inflated balloon, as well as the normal force. Therefore, to obtain the most accurate value, actual human digital nerves were used in the pulling experiments. One end of the nerve was gripped by the Instron metallic gripper and the other end was gripped by the surgical gripper, which was positioned by a clamp locked by a rigid multi-joint arm (as shown in Figure 10).

After the soft pneumatic channel was inflated with the specified air pressure, the digital nerve was stretched at a rate of 1 mm/second until grip failure occurred. Grip failure at the instrument–tissue interface includes tissue slippage through the gripping area and tissue tearing. Unlike traditional forceps with teeth or wave patterns, which have stress concentration points or edges, the inflated balloon of the proposed surgical gripper provides distributed force to the gripped nerve. Thus, only tissue slippage will occur during the pulling experiments. We investigated the pulling force at various pressures ranging from 90 to 180 kPa with a step of 30 kPa. The peak force was sensed and recorded by the load cell integrated with the Instron metallic gripper. The experiment was repeated five times for each pressure. The digital nerves were thus cut into 20 segments of 2 cm length, and a new digital nerve sample was used for each experiment. The average gauge length in the pulling experiments was 7.27 mm. The experimental results of the peak force measurement are summarized in Table 1. Based on the experimental results, the pulling force did not elevate substantially when the air pressure was increased from 150 to 180 kPa. Additionally, it is important to note the influence of the nerve dimension and gripped position on the pulling force.

### 3.2. Deformation Measurement Experiments

**Figure 11:**
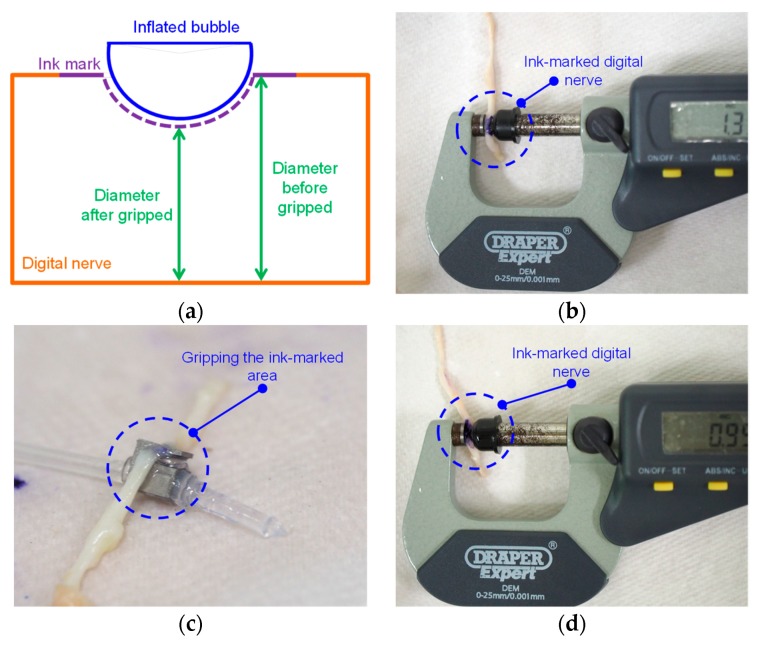
The deformation measurement experiments on digital nerves: (**a**) the schematic diagram for the deformation measurement experiments; (**b**) measuring the original diameter of the ink-marked area; (**c**) gripping the ink-marked area using the proposed surgical gripper; and (**d**) measuring the diameter of the ink-marked area after being gripped.

The schematic diagram of measuring the deformation gripped by the proposed surgical gripper is shown in Figure 11a. An ink marking was made on the surface of the section of the digital nerve that was required to be gripped. The original diameter of the ink-marked section was measured and recorded (as shown in Figure 11b). The proposed surgical gripper was then used to grip the ink-marked area, and the compression was held for 10 s (as shown in Figure 11c). Afterwards, the diameter of the gripped ink-marked section was measured and recorded again (as shown in Figure 11d).

In order to avoid bias from the experimenters, two experienced surgeons were asked to measure the diameter variation of the digital nerve. The original diameter of the digital nerve was measured by one surgeon, and the other surgeon was asked to measure the diameter of the digital nerve after it was gripped by the proposed surgical gripper. Each measurement was repeated five times, and the average value was recorded as the measurement result, with the purpose of reducing measurement error. Pressures ranging from 90 to 180 kPa with a step of 30 kPa were respectively applied to soft inflatable actuator, and each setting was repeated three times (e.g., 90 kPa for 10s on the digital nerve was repeated three times, each at a different section). The differences in the diameter before and after being gripped based on different pressures are summarized in Table 2. The results suggest that deformation depends on the original diameter and the gripped location of the digital nerve. Thus, the deformity could vary even under the same pressure setting. It is observed that the average deformation of a digital nerve with an average diameter of 1.45 mm is less than 0.22 mm. Therefore, the average deformity is less than 15% of the original diameter.

### 3.3. Gripping Tests

In order to validate the proposed surgical gripper’s abilities to manipulate a digital nerve during repair surgery, gripping tests were conducted based on a digital nerve located in the middle finger of a cadaver.

**Figure 12:**
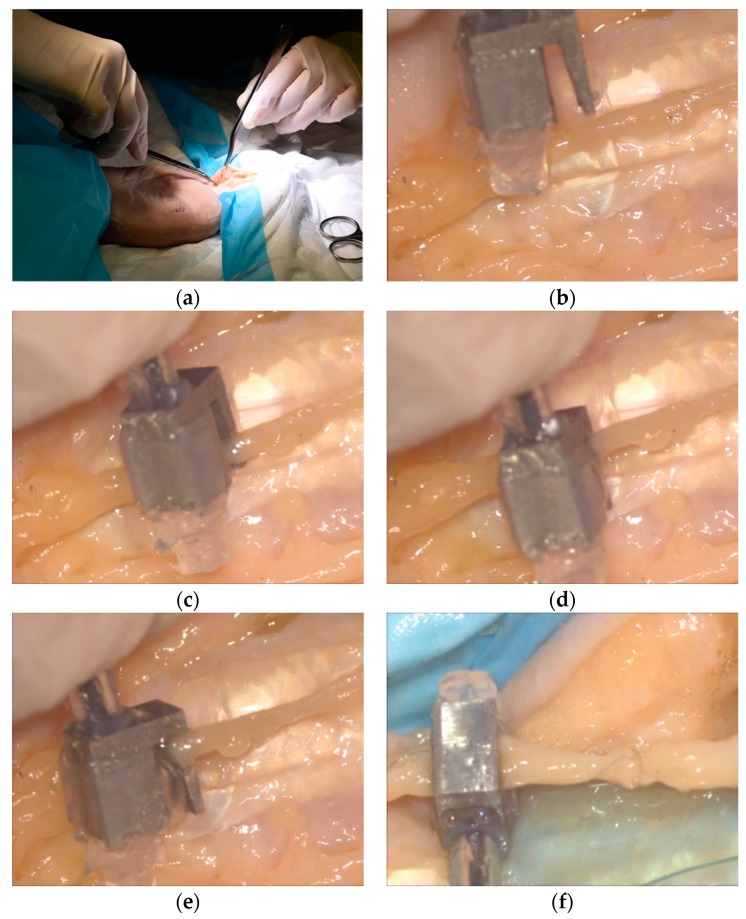
Gripping experiment on a digital nerve located in the middle finger of a cadaver using the proposed soft hybrid surgical gripper ((**b**–**f**) were captured from microscopic view): (**a**) making an incision to expose the digital nerve of the middle finger; (**b**) approaching the digital nerve that needed to be gripped; (**c**) scooping up the digital nerve; (**d**) inflating the soft pneumatic actuator to grip the digital nerve; (**e**) the zoomed-in figure of the inflated balloon; and (**f**) rotating the digital nerve for suturing the reverse side of the digital nerve and for observation.

First, the surgeon made an incision in the middle finger to expose the digital nerve with sharp dissection (as shown in Figure 12a). The following operations on the digital nerve were conducted under a microscope, and the figures were captured from the microscopic view. The proposed soft hybrid surgical gripper was manually controlled to approach the section of the digital nerve that needed to be gripped (as shown in Figure 12b). The digital nerve was then scooped up from the surrounding tissues (as shown in Figure 12c). After that, the soft pneumatic actuator was pressurized to firmly grip the digital nerve by the inflated balloon (as shown in Figure 12d). Then, 150 kPa was selected to inflate the soft pneumatic actuator during the gripping experiments according to the measured contact and pulling force in the force sensing experiments. The interaction between the balloon and the gripped nerve is shown in Figure 12e. Finally, the operation rotating the digital nerve was tested (as shown in Figure 12f), which is used for suturing the reverse side of the digital nerve and for observation.

Based on the experimental results, our findings show that the overall dimension of the proposed surgical gripper can allow for the manipulation of the digital nerve within the limited working space. In addition, the hook retractor structure of the proposed surgical gripper can scoop up the digital nerve from the surrounding tissues and hold it in place by the inflated balloon.

## 4. Discussion and Conclusions

In this paper, a hybrid soft robotic surgical gripper is proposed for delicate nerve manipulation in digital nerve repair surgery. It combines a soft inflatable actuator and a gripper shell with a hook-shaped structure. The soft pneumatic actuator is inflated to achieve soft contacts and compliant grip, which potentially reduces the risk of over-gripping damage. It also facilitates the surgeon’s delicate nerve manipulation process. The surgeon does not have to worry about sudden over-gripping force stimulation, as the gripping force generated by the proposed surgical gripper will vary little when steady air pressure is provided. In contrast, handling a digital nerve by using traditional forceps requires extreme caution. Additionally, the rigid hook retractor allows surgeons to scoop up the digital nerve from the surrounding tissues. Contact/pulling force sensing experiments and deformation measurement experiments were conducted to evaluate the performance of the proposed surgical gripper. The operability of the proposed surgical gripper used in digital repair surgery was tested based on cadaver experiments.

Furthermore, this new design possesses other key qualities, such as having low-cost components and being water-resistant and non-corrosive. The cost for each gripper is approximately $25 US dollars ($10 US dollars for the soft inflatable actuator and $15 US dollars for the fabrication of the gripper shell). The proposed fabrication method is also simple, low cost, and effective. The communicating vessels-based fabrication can prevent the formation of small bubbles in the soft pneumatic channels and can be easily extended to other applications. The air-supply tube combined with an interior steel wire forms a flexible arm, which not only supplies air pressure, but also provides easy bending into any desired configuration during digital nerve manipulation procedures. Moreover, the fabricated soft inflatable actuator is a pluggable component in the proposed soft hybrid surgical gripper. The soft inflatable actuator is designed for one-time use and can be thrown away after surgery. The stainless-steel hook retractor can be sterilized before and after surgery; hence, it can be reused many times. A new inflatable actuator can be inserted into the nest of the gripper for the next surgery.

**Figure 13:**
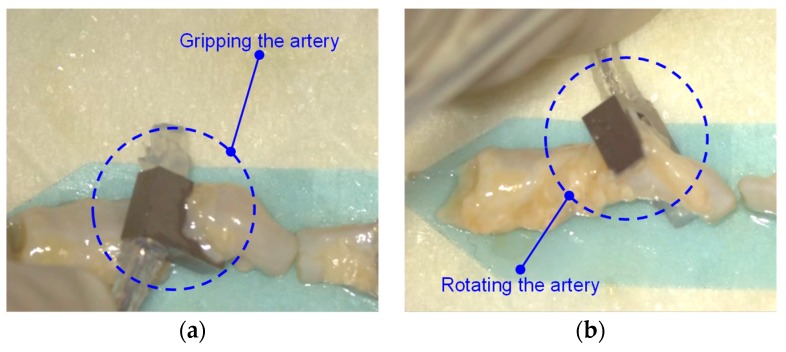
Gripping experiment on an artery using the soft hybrid surgical gripper (the figures were captured from microscopic view): (**a**) gripping the artery using the inflated balloon; (**b**) rotating the artery for observation.

Additionally, the proposed surgical gripper can be extended to grip tissues of larger dimensions in other surgeries by enlarging the size of the hook structure. We revised the design of the hook structure by increasing it 2 mm (the other parts remained the same) for gripping a human artery. It can also grip an artery via the inflated balloon (as shown in Figure 13). In the current design, the new surgical gripper includes a rigid hook-shaped structure in order to provide larger gripping force and to scoop up the digital nerve from the surrounding tissues. In future studies, a fully soft robotic gripper will be proposed for digital nerve repair surgery.

## Figures and Tables

**Table 1:** Peak force (N) for the pulling experiments.

Pressure No.	90 kPa	120 kPa	150 kPa	180 kPa
1	0.21	0.32	0.58	0.56
2	0.17	0.33	0.41	0.46
3	0.29	0.29	0.51	0.62
4	0.25	0.29	0.54	0.47
5	0.17	0.32	0.48	0.5
Average	0.218	0.31	0.504	0.522

**Table 2:** Results of the deformation experiments.

Pressure	No.	Diameter before Gripped (mm)	Diameter after Gripped (mm)	Difference (mm)/Deformity (%)
90 kPa	1	1.23	1.10	0.13/11%
	2	1.46	1.28	0.18/12%
	3	1.30	1.10	0.2/15%
120 kPa	1	1.52	1.33	0.19/13%
	2	1.69	1.59	0.1/6%
	3	1.60	1.21	0.39/24%
150 kPa	1	1.90	1.59	0.31/16%
	2	1.13	1.00	0.13/12%
	3	1.48	1.15	0.33/22%
180 kPa	1	1.30	1.00	0.3/23%
	2	1.08	0.97	0.11/10%
	3	1.72	1.47	0.25/15%
